# Supplementary material for: Allosteric Coupling in Full-Length Lyn Kinase Revealed by Molecular Dynamics and Network Analysis
Source: Int J Mol Sci. 2025 Jun 18;26(12):5835. doi: 10.3390/ijms26125835 (PMC12192854; doi:10.3390/ijms26125835)
Supplement: Supplementary file 1 [file ijms-26-05835-s001.zip › SI_figures.pdf]

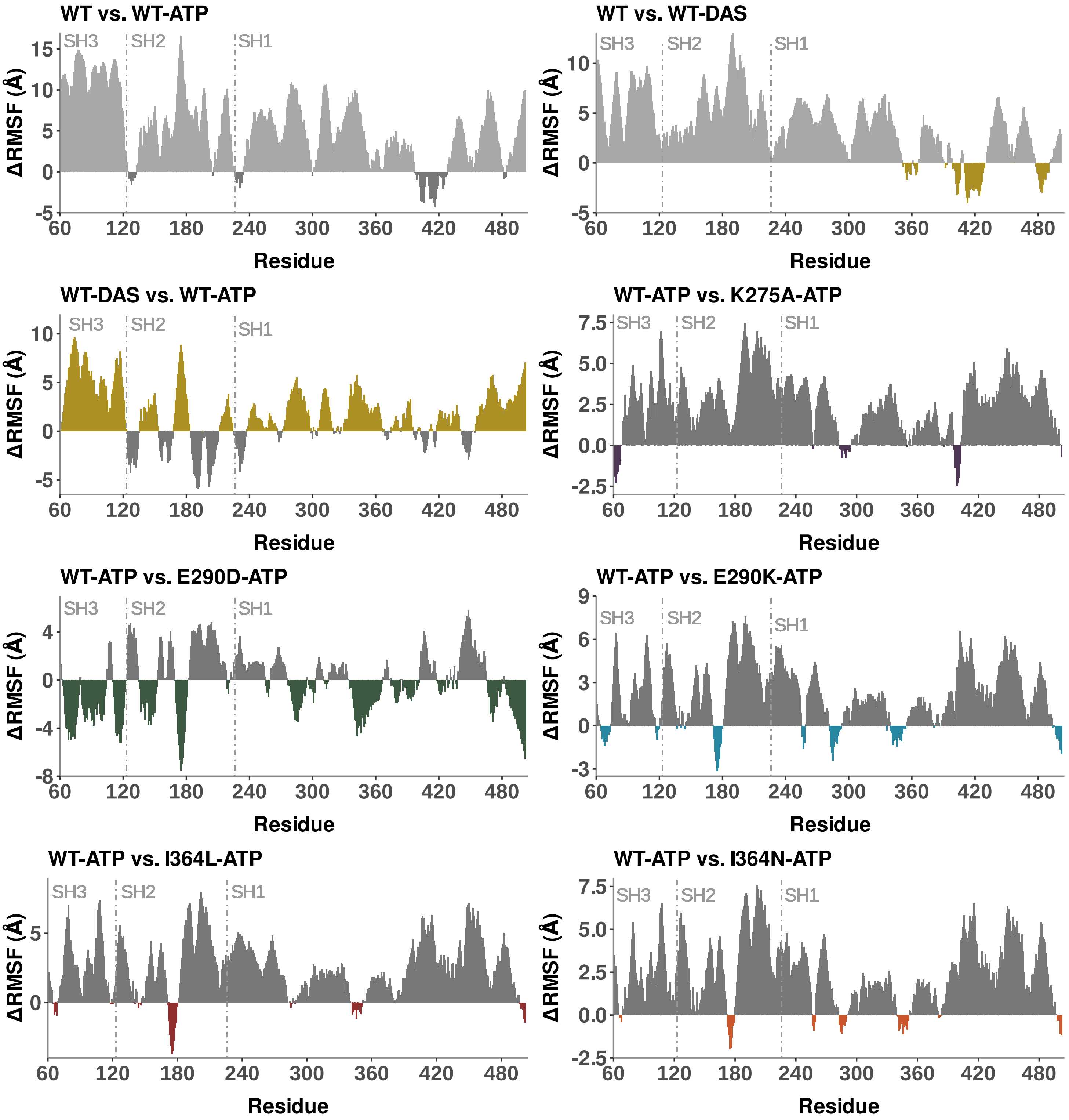

Figure S1. Comparative residue-level flexibility changes across systems. ΔRMSF plots showing the difference in Cα atomic fluctuations between selected pairs of systems. Each panel compares WT or WT-ATP against ligand-bound or mutant systems, with values calculated as ΔRMSF = reference − comparison. Positive values indicate higher flexibility in the reference system. Domain boundaries (SH3, SH2, SH1) are marked by vertical dashed lines.

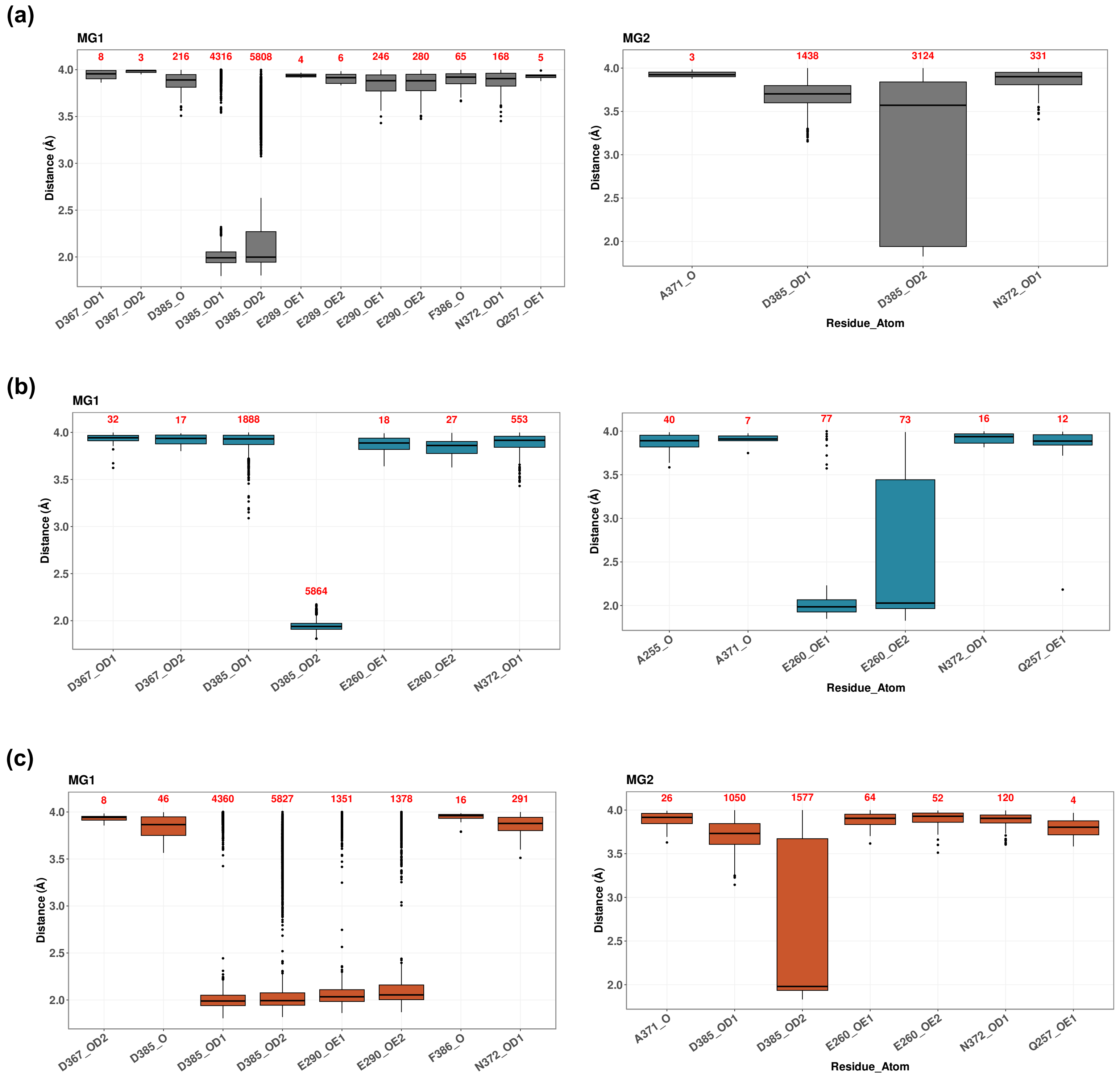

Figure S2. Distances between magnesium ions and coordinating residues across selected systems. Boxplots represent distances (in Å) between magnesium ions MG1 (left panels) and MG2 (right panels) and coordinating atoms across MD trajectories for **(a)** WT-ATP, **(b)** E290K-ATP, and **(c)** I364N-ATP systems. Red numbers above each box indicate the number of trajectory frames in which the corresponding interaction was observed. WT-ATP shows tight and consistent coordination, particularly involving D385 and water-mediated contacts. In contrast, mutant systems exhibit disrupted or asymmetric coordination patterns, particularly at MG2, consistent with impaired catalytic geometry in kinase-inactive conformations.

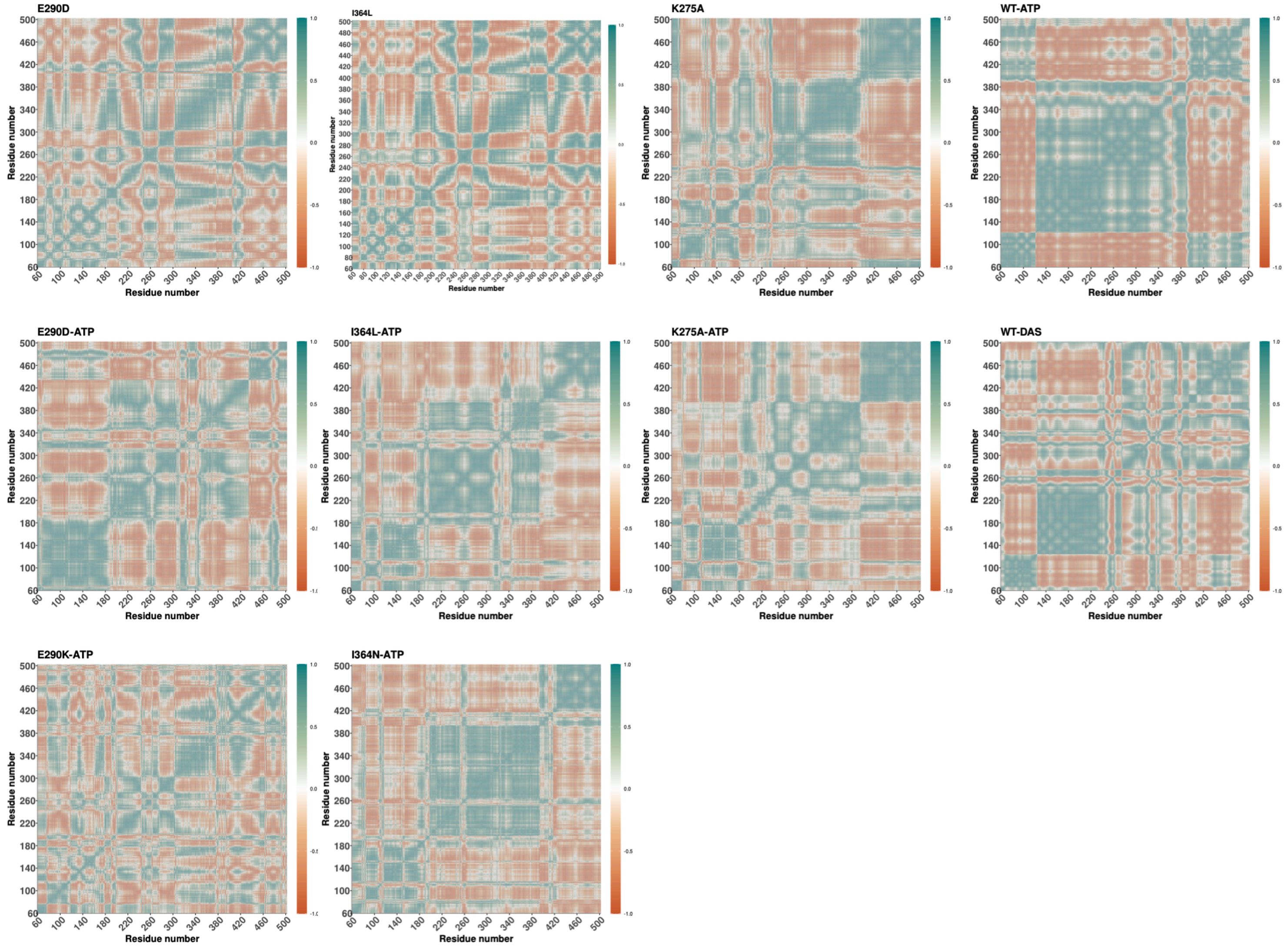

Figure S3. Dynamical cross-correlation matrices (DCCMs) for 10 Lyn kinase systems. Each matrix shows pairwise Cα atom correlations over the simulation trajectory, with positive correlations (teal) indicating coordinated motions and negative correlations (red) indicating anti-correlated movements. Compared to WT-ATP, which displays structured long-range correlations across domains, mutant and inhibitor-bound systems exhibit varying degrees of correlation loss and fragmentation. These maps complement the main figure by providing a full comparison across the remaining systems. DCCMs for WT, E290K, and I364N are shown in Figure 5.

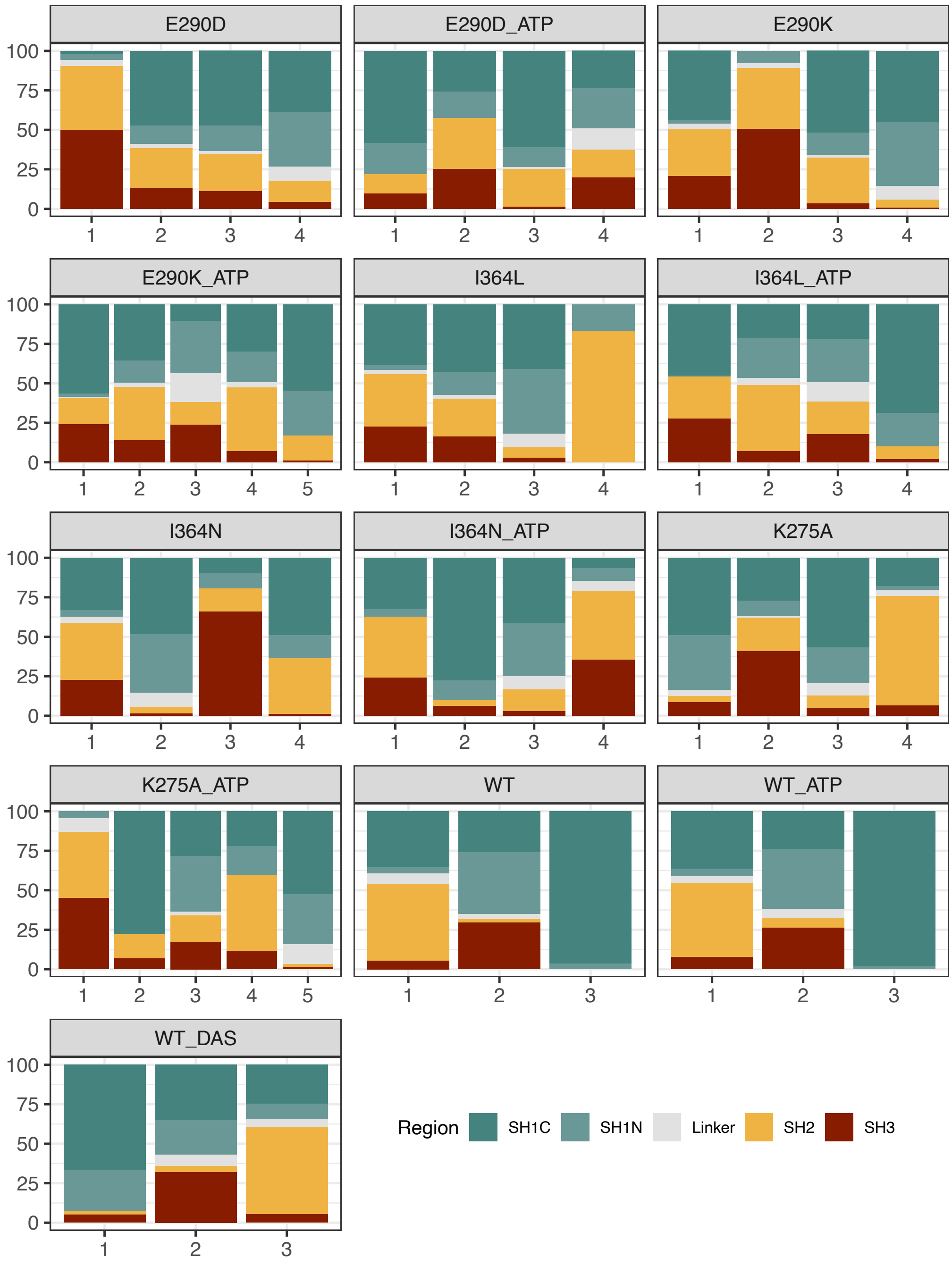

Figure S4. Module composition and structural region purity across all systems. Stacked bar plots show the distribution of structural regions (SH3, SH2, linker, SH1N, SH1C) within each detected community module per system, as identified by Louvain clustering on dynamic correlation networks. Each bar corresponds to a distinct module, and region-specific colors indicate the degree of compositional heterogeneity. Low-purity modules (i.e., those with contributions from multiple domains) reflect dynamically integrated communities relevant for long-range allosteric communication.

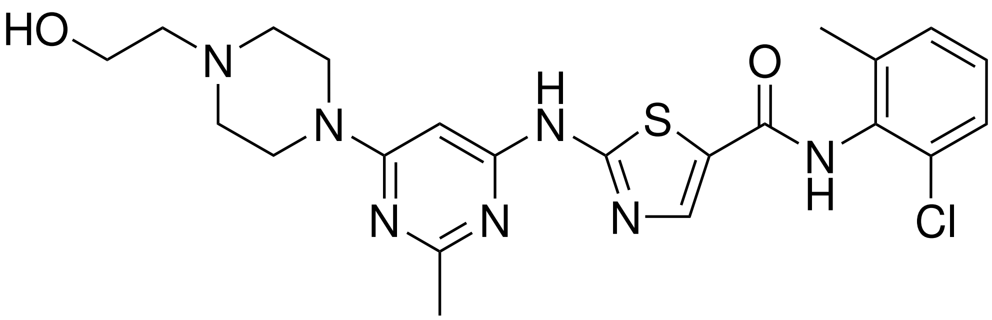

Figure S5. Chemical structure of dasatinib, an ATP-competitive kinase inhibitor. The molecule has a molecular formula of $C_{22}H_{26}ClN_7O_2S$. The compound features a thiazole core, substituted aniline moiety, and heterocyclic linkers that mediate π-stacking and van der Waals interactions in the ATP-binding pocket of kinases.
