# Supplementary material for: Allosteric Coupling in Full-Length Lyn Kinase Revealed by Molecular Dynamics and Network Analysis
Source: Int J Mol Sci. 2025 Jun 18;26(12):5835. doi: 10.3390/ijms26125835 (PMC12192854; doi:10.3390/ijms26125835)
Supplement: Supplementary file 1 [file ijms-26-05835-s001.zip › SI_videos.pdf]

Supplementary Video S1. Comparison of full-length Lyn kinase dynamics in WT and I364N apo systems. Structures from MD trajectories are aligned and visualize overall conformational behavior. WT (gray) and I364N (orange) are shown highlighting differences in mobility. In the WT system, the SH3 domain undergoes opening and outward displacement, consistent with an active-like ensemble. In contrast, I364N exhibits restricted SH3 mobility and a more compact conformation, reflecting impaired regulatory displacement. These differences are based on the highest motions extracted from the trajectories.

Supplementary Video S2. ATP binding and activation loop dynamics in the WT-ATP system. This video shows representative frames from a 6 µs MD trajectory of full-length Lyn kinase in its ATP-bound form. Frames were extracted every 10 ns to illustrate progressive conformational changes. All over the simulation, ATP remains stably coordinated in the binding site, while the activation loop (A-loop) undergoes distinct rearrangements, reflecting its conformational plasticity during catalysis. Movements of the SH3 domain and interdomain linkers are also visible, highlighting coordinated transitions consistent with an active-like state.
